# Update of Genetic Diversity of Porcine Circovirus Type 2 in Chile Evidences the Emergence of PCV2d Genotype

**DOI:** 10.3389/fvets.2021.789491

**Published:** 2021-12-17

**Authors:** Naomi Ariyama, Belén Agüero, Valentina Valdés, Felipe Berrios, Sergio Bucarey, Sunil Mor, Barbara Brito, Victor Neira

**Affiliations:** ^1^Departamento de Medicina Preventiva Animal, Facultad de Ciencias Veterinarias y Pecuarias, Universidad de Chile, Santiago, Chile; ^2^Departamento de Ciencias Biológicas, Facultad de Ciencias Veterinarias y Pecuarias, Universidad de Chile, Santiago, Chile; ^3^Veterinary Population Medicine Department, College of Veterinary Medicine, University of Minnesota, St. Paul, MN, United States; ^4^The iThree Institute, University of Technology Sydney, Sydney, NSW, Australia

**Keywords:** PCV2, genetic diversity, PCV2 genotypes, Chile, genotype shift

## Abstract

Porcine Circovirus 2 (PCV2) can cause multiple clinical conditions known as porcine circovirus-associated diseases (PCVAD). Before the wide availability of PCV2 vaccines, PCVAD resulted in significant losses to the global swine industry. PCV2's rapid evolutionary dynamics are comparable to single-stranded RNA viruses. Thus, shifts in the dominance and distribution of different genotypes may frequently occur, resulting in the emergence and spread of varying PCV2 genotypes and recombinant strains in swine. This study aims at identifying the PCV2 genotypes currently circulating in Chile. Seven hundred thirty-eight samples were obtained from 21 swine farms between 2020 and 2021. The samples were tested using PCR for species detection and genotyping. Sequencing and phylogenetic analyses were conducted in selected samples. PCV2 was detected in 26.9% of the PCR reactions and 67% of the sampled farms. The genotypes were determined in nine farms, PCV2a in one farm, PCV2b in four, and PCV2d in five, with PCV2b and PCV2d co-circulating in one farm. The phylogenetic analysis of twelve ORF2 sequences obtained (PCV2a = 5; PCV2b = 4; PCV2d = 3) showed a PCV2a Chilean strains monophyletic cluster; closely related to Chilean viruses collected in 2012 and 2013. Of the three different PCV2b sequenced viruses, two viruses were close to the root of the PCV2b group, whereas the remaining one grouped with a South Korean virus. PCV2d sequences were closely related to Asian viruses. A previously reported PCV2a/PCV2d recombinant strain was not detected in this study. Our results suggest the emergence and potential shift to PCV2d genotype in Chilean farms.

## Introduction

Porcine circoviruses (PCVs) are small (12–23 nm), non-enveloped, icosahedral, circular single-stranded DNA viruses (1). Porcine Circovirus 2 (PCV2) is the most studied PCVs species due to its pathogenic potential, associated with multiple clinical conditions (2). These are known as porcine circovirus-associated diseases (PCVAD); including the PCV2 systemic disease, formerly post-weaning multisystemic wasting syndrome (PMWS), and diverse respiratory, reproductive, intestinal, and neurological syndromes (3–5). Although nowadays, the most common PCV2 manifestation is subclinical (5). Currently, PCV2 is widely distributed in domestic and wild swine, negatively affecting pork production (1, 2). Before the availability of vaccines, PCVAD was a major problem for the global swine industry. When uncontrolled, PCV2 losses were estimated at £88 million/year in England only, with subclinical infections causing higher costs than clinical manifestations (5, 6).

PCV2 exhibits evolutionary dynamics similar to RNA viruses, with a nucleotide substitution rate of 1.2 × 10^−3^ substitutions/site/year and frequent recombination events (7, 8). According to this and the growing number of viral sequences, PCV2 genotype classification is continually changing, and the latest genotyping method assigned eight genotypes: PCV2a to PCV2h (9). Periodic waves of PCV2 genotypes or “genotype shifts” have affected swine; this also occurs in some PCV2 recombinant viruses (8, 10). Initially, the most frequent genotype in affected pigs was PCV2a until the 2000 s, then outclassed by PCV2b and afterward by PCV2d (1). Additionally, the local and global movement of sub-clinical animals allowed the rapid PCV2 spread (7, 8). The remaining genotypes have limited virulence or distribution (8, 9).

The first official detection of PCV2 in Chile occurred in 2006 (11). Between 2005 and 2013, Neira et al. reported the high occurrence of PCV2b (52%) and a PCV2a/PCV2d recombinant lineage (34%) in Chilean swine intensive farms (10). Monitoring the circulating genotypes is critical to understand changes that could lead to lower vaccine protection or higher occurrence of clinical or subclinical disease. Due to the rapid emergence and spread of PCV2 genotypes, this study aims to review the current distribution of PCV2 genetic types in Chile.

## Materials and Methods

### Samples

The samples were obtained from submissions for PCV2 diagnostic at Universidad de Chile Animal Virology Laboratory between March 2020 to July 2021. The samples were originated for the diagnosis of animals with suspected clinical PCVAD (69) or active molecular surveillance of PCV2 (669). A total of 640 sera, 92 oral fluids, and 6 lung samples were included in this study. Samples were obtained from 21 commercial swine farms (A-U), which represent >90% of the commercial swine companies in the country. Swine farms are located in Valparaiso, Metropolitan, O'Higgins, Maule, and Ñuble regions in Central Chile (Table 1, Supplementary Figure 1). All farms had an active vaccination program against PCV2 in piglets, and in general, did not evidence clinical PCVAD during the sampling. Sera were collected from at least 16 pigs aged 10–20 weeks old in each farm. The oral fluids were collected at a similar age. Samples were stored at 4°C until processing at Animal Virology Laboratory, Facultad de Ciencias Veterinarias y Pecuarias, Universidad de Chile. Sample collection is summarized in Table 1.

**Table 1:** Summary of sample collection and PCR results.

					**qPCR**			**PCR Genotyping**		
**Region**	**Farm ID**	**Sample**	**N samples**	**N pooled samples**	**Tested**	**Positive**	**%**	**PCV2a**	**PCV2b**	**PCV2d**
O'Higgins	Farm A	Serum	32	8	8	0	0.0	ND		
Maule	Farm B	Serum	32	8	8	0	0.0	ND		
		Oral Fluid	8	-	8	1	12.5	ND		
Metropolitana	Farm C	Serum	64	16	16	8	50.0	2	0	1
		Oral fluid	5	-	5	4	80.0	ND		
		Lung	2	-	2	2	100.0	2	0	1
Metropolitana	Farm D	Oral fluid	12	-	12	4	33.3	2	0	0
Ñuble	Farm E	Serum	32	8	8	0	0.0	ND		
		Oral fluid	4	-	4	0	0.0	ND		
O'Higgins	Farm F	Serum	10	-	10	4	40.0	ND		
		Oral fluid	8	-	8	0	0.0	ND		
O'Higgins	Farm G	Oral fluid	4	-	4	4	100.0	0	0	1
		Serum	140	35	35	16	45.7	ND		
Maule	Farm H	Serum	32	8	8	2	25.0	ND		
		Oral fluid	5	-	5	1	20.0	ND		
Bio Bio	Farm I	Serum	32	8	8	1	12.5	ND		
O'Higgins	Farm J	Serum	32	8	8	1	12.5	1	0	0
Maule	Farm K	Serum	32	8	8	0	0.0	ND		
		Oral fluid	3	-	3	0	0.0	ND		
O'Higgins	Farm L	Serum	44	8	20	3	15.0	ND		
		Oral fluid	4	-	4	3	75.0	0	2	0
O'Higgins	Farm M	Serum	32	8	8	0	0.0	ND		
		Oral Fluid	4	-	4	1	25.0	1	0	0
Valparaiso	Farm N	Serum	16	4	4	0	0.0	ND		
Metropolitana	Farm O	Serum	16	4	4	1	25.0	ND		
Metropolitana	Farm P	Serum	16	4	4	0	0.0	ND		
O'Higgins	Farm Q	Serum	16	4	4	1	25.0	0	0	1
O'Higgins	Farm R	Serum	16	4	4	0	0.0	ND		
Maule	Farm S	Serum	16	4	4	0	0.0	ND		
O'Higgins	Farm T	Serum	18	-	18	3	16.7	ND		
		Oral fluid	35	-	35	7	20.0	ND		
		Lung	4	-	4	3	75.0	0	0	2
O'Higgins	Farm U	Serum	12	-	12	10	83.3	0	0	2
Total	21		738	147	297	80	26.9	8	2	8

*ND, Not determined*.

### Testing and Sequencing

The samples were tested by real-time PCR, genotyped, and sequenced. Briefly, samples were centrifugated at 2,500 g for 10 min. The DNA was extracted individually in lungs and oral fluids samples, whereas sera were pooled (4 samples per pool) before DNA extraction. The extraction was performed using PureLink Genomic DNA Mini Kit (Invitrogen, K182001, CA, USA) following the manufacturer's instructions. All samples were tested by real-time PCR and Ct <40 was considered positive (12). A genotype-specific PCR previously described was used to identify PCV2a, PCV2b, and PCV2d in samples where real-time PCR was Ct < 35 (13). Additionally, in 15 samples selected based on Ct value per case (Ct range: 11.8 to 39.4) ORF2 sequencing was attempted at Veterinary Diagnostic Laboratory, University of Minnesota (12).

### Phylogenetic Analysis

Sequences were aligned with publicly available reference sequences from all PCV2 genotypes (a-h) using MUSCLE (14). Two phylogenetic analyses were performed: one including ORF2 partial and complete sequences from Chile (excluding recombinants) and another with only complete ORF2 sequences. The Maximum likelihood (ML) tree was estimated using RAxML (15) with 1,000 bootstrap replications, in Geneious Prime 2021.2 (16) and then visualized in Figtree (http://tree.bio.ed.ac.uk/software/figtree/).

## Results and Discussion

Overall results are summarized in Table 1. PCV2 was detected in 14 out of 21 farms, distributed in all the regions in the study, except for Ñuble and Valparaiso. This geographic distribution is similar to previous results (10). Eighty out of 297 PCR reactions (26.9%) were positive for PCV2, corresponding to 25/92 oral fluids (27.2%), 50/199 pooled serum samples (25.1%), and 5/6 lung samples (83.3%). During the study, PCV2 was predominantly detected in oral fluids from 100 to 120 days-old pigs, with and without apparent clinical signs. These results agree with a previous study, where PCV2 detection was higher in oral fluids compared to serum samples from 14 to 16 weeks-old pigs (17). In 19 samples with Ct < 35, belonging to 13 positive farms, PCR genotyping was attempted. Two viruses were identified as PCV2a, eight as PCV2b, eight as PCV2d, and nine samples did not amplify, probably due to the low quantity/quality of PCV2 genetic material. Thus, in nine out of 13 farms at least one of the circulating genotypes was identified. In one farm, two PCV2 genotypes (PCV2b and PCV2d) were identified.

**Figure 1:**
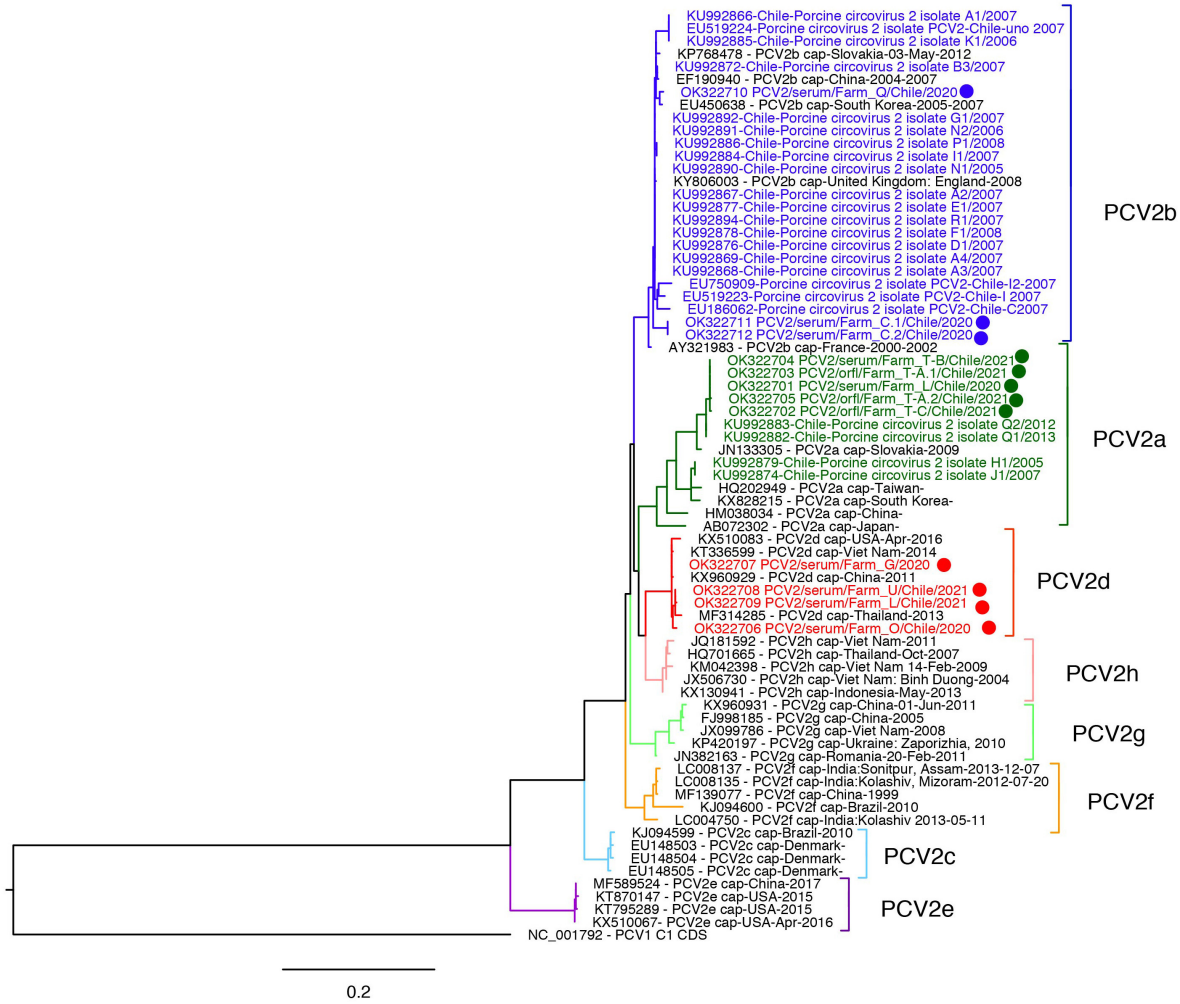
Maximum likelihood phylogenetic tree of Porcine circovirus 2 ORF2 sequences from Chile. The analysis involved 72 sequences, 36 corresponded to PCV2 genotypes (a-h) reference sequences, sequences from Chile from 2005 to 2013 (colored), and sequences from this study (dots). PCV1 (NC001792) was incorporated as an outgroup.

Sequencing was successful in 12 samples from 11 different farms, sequences were deposited in GenBank (Accession numbers OK322701-OK322712). The ORF2 sequencing was successful in all samples with Ct < 30. The phylogenetic tree grouped five isolates into the PCV2a cluster, four in PCV2d, and three in PCV2b clusters (Figure 1, Supplementary Figure 2). Sequences belonging to PCV2a and PCV2d were collected from 4 farms each, and PCV2b sequences from two farms. The PCV2a isolates are grouped in a monophyletic cluster with PCV2a isolates sequenced in Chile in 2012–2013 and are 2.00–6.85% different to reference PCV2a sequences. Interestingly, the current observed PCV2a Chilean isolates were collected from farms related geographically and by pig flow. Two of the three PCV2b sequences were close to the root of the PCV2b genotype cluster, and the remaining one grouped with a sequence from South Korea (EU450638). On the other hand, PCV2d sequences were highly similar (99.43% identity) and grouped with Asian viruses. In contrast with PCV2a, PCV2d viruses were collected from three unrelated farms. Interestingly, we did not detect the PCV2a/PCV2d recombinant strain detected in Chile in a previous study. However, considering the low number of samples sequenced, the current circulation of this recombinant strain cannot be ruled out.

According to a previous report, the PCV2 genotypes occurrence was: 52% of PCV2b from 14 out of 20 intensive farms, 35% of a PCV2a/PCV2d recombinant lineage from seven farms, and 14% of PCV2a from three farms (10). Here, the recombinant strains were not detected, and PCV2b was detected in four farms and successfully sequenced only in two. Also, we observed the emergence of PCV2d and the maintenance of the PCV2a genotype.

In this study, we observed some limitations, essentially the lower detection of PCV2 and the low number of sequences obtained. In this sense, the distribution and occurrence of the detected genotypes may be underestimated in our results. Neira et al. (10), performed the sequencing from PCV2 positive tissues collected from PMWS/PCVAD-affected pigs, but nowadays is difficult to observe the clinical symptoms of PCV2. The lower presence of PMWS/PCVAD-affected pigs is most likely the result of vaccination protocols performed at Chilean farms (11). Vaccination also reduces viremia and shedding during PCV2 infection, limiting viral detection (18).

Overall results of detection and sequencing suggest the potential genotype shift to PCV2d in Chile, although the low number of genotyped samples restricts this conclusion. Similar findings have been reported in recent years in several countries such as the USA, Korea, and Austria (13, 19, 20). This genotype shift may be influenced by natural selection but also by vaccination pressure (21, 22). In this sense, widely administered PCV2 vaccines in Chilean swine since 2008 may have contributed to PCV2 evolution. As previously mentioned, PCV2 genotype shift has been reported in several countries. However, in South America, PCV2d has been reported on three occasions: one during an outbreak of PMWS/PCVAD in vaccinated pigs from Brazil, that suggested a vaccine failure; another from vaccinated and unvaccinated pigs from Uruguay; and the last from unvaccinated pigs with and without clinical signs in Colombia (23–25). Further research on these viruses is necessary to understand the impact of these genotype shifts in the swine industry.

In conclusion, we did not detect a previously reported prevalent PCV2a/PCV2d recombinant strain. Our results suggest the emergence and potential shift to PCV2d genotype in Chilean farms, as in other parts of the world, highlighting the rapid evolution of PCV2 and the relevance of pathogen surveillance and monitoring to the early detection of new genotypes.

## Data Availability Statement

The original contributions presented in the study are included in the article/Supplementary Materials, further inquiries can be directed to the corresponding author.

## Ethics Statement

Ethical review and approval was not required for the animal study because samples were obtained from clinical cases by veterinary practitioners; therefore, ethical approval was not considered. Written informed consent for participation was not obtained from the owners because samples were obtained from clinical cases by veterinary practitioners; therefore, consent forms were not considered.

## Author Contributions

NA, FB, BA, and VN: study design and conceptualization. SB and VN: funding and resources. BA, FB, and VV: samples collection and processing. BA, FB, VV, BB, and SM: performed the essays. NA, BB, and VN: data analysis and NA, BA, BB, and VN: wrote the paper. All authors critically evaluated the paper.

## Funding

This study was partially funded by the Animal Virology Laboratory, Facultad de Ciencias Veterinarias y Pecuarias, Universidad de Chile; Programa Fondecyt N° 11170877 and N° 1211517 to VN; Programa Fondef N° ID19I10135 to SB and VN.

## Conflict of Interest

The authors declare that the research was conducted in the absence of any commercial or financial relationships that could be construed as a potential conflict of interest.

## Publisher's Note

All claims expressed in this article are solely those of the authors and do not necessarily represent those of their affiliated organizations, or those of the publisher, the editors and the reviewers. Any product that may be evaluated in this article, or claim that may be made by its manufacturer, is not guaranteed or endorsed by the publisher.
