# Supplementary material for: Update of Genetic Diversity of Porcine Circovirus Type 2 in Chile Evidences the Emergence of PCV2d Genotype
Source: Front Vet Sci. 2021 Dec 17;8:789491. doi: 10.3389/fvets.2021.789491 (PMC8718606; doi:10.3389/fvets.2021.789491)
Supplement: Supplementary file 1 [file Data_Sheet_1.PDF]

Supplementary Material

1 Supplementary Figures

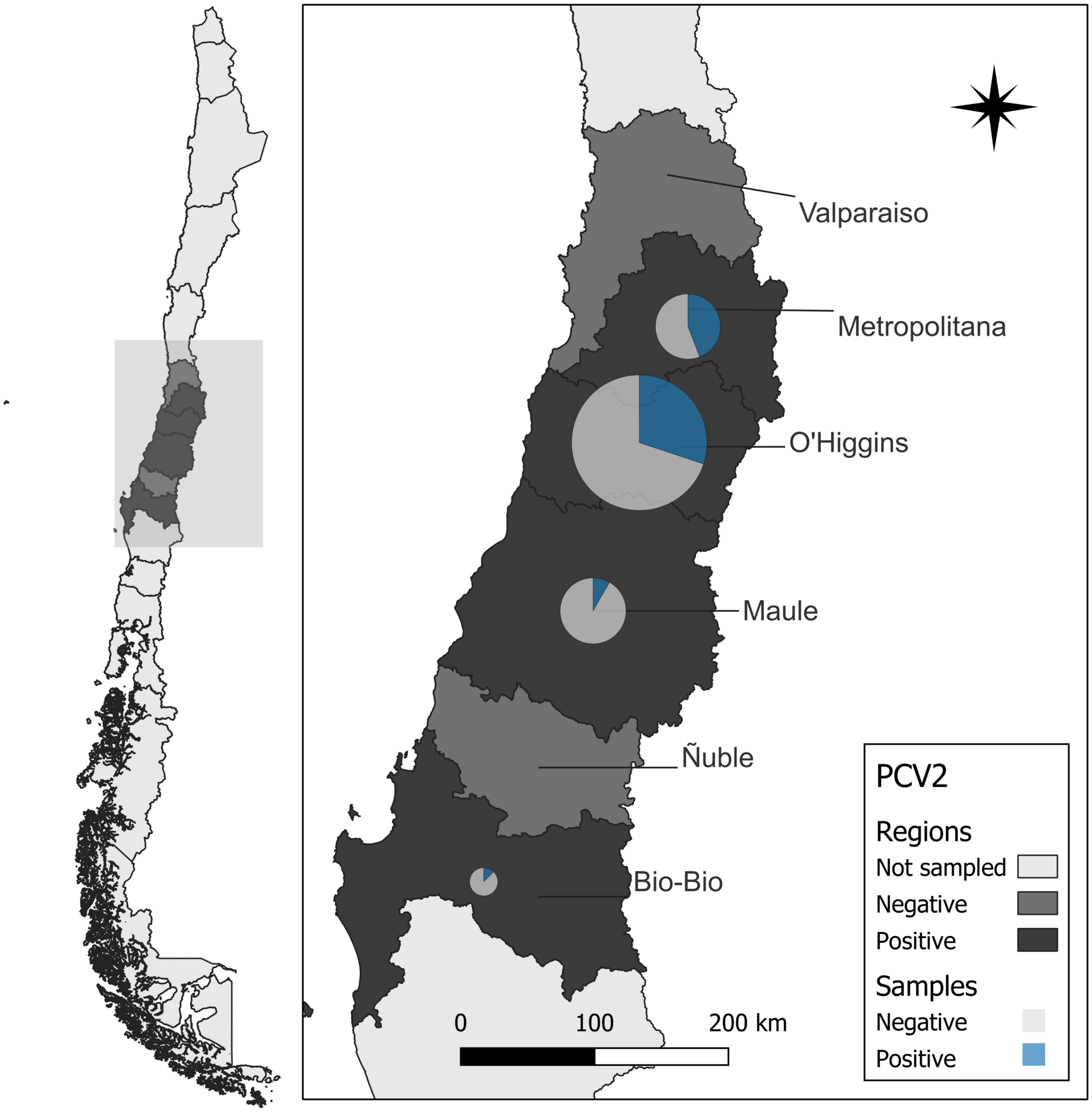

Supplementary Figure 1. Overall results of PCV2 detection by Regions of Chile.

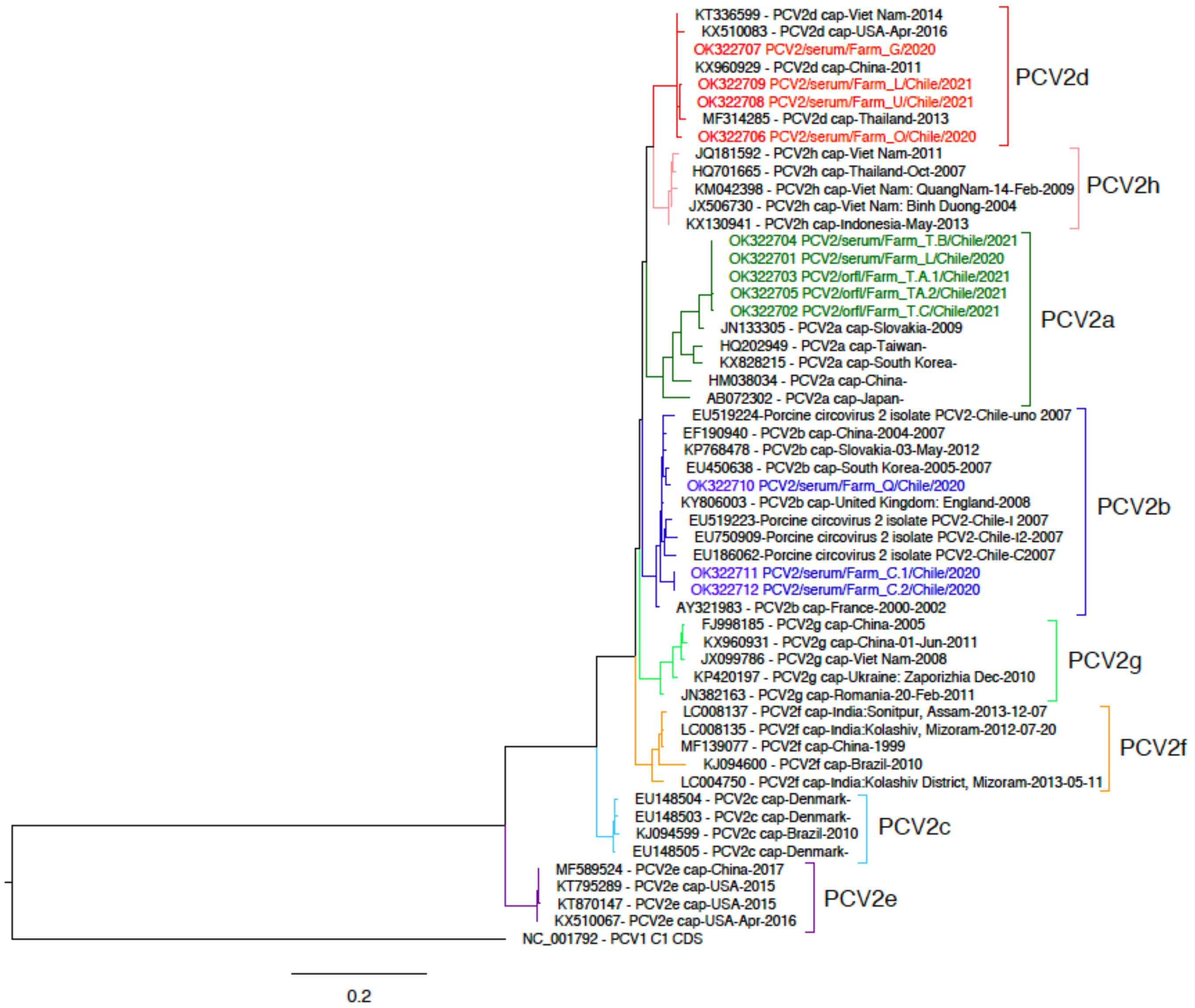

Supplementary Figure 2. Maximum likelihood phylogenetic tree of Porcine circovirus 2 complete ORF2 sequences from Chile. The analysis involved 53 sequences, 36 corresponded to reference sequences of all PCV2 genotypes (a-h), four to other sequences from Chile, 12 from this study (colored), and PCV1 (NC001792) was the outgroup.
